# Loss of interest, depressed mood and impact on the quality of life: Cross-sectional survey

**DOI:** 10.1186/1471-2458-11-826

**Published:** 2011-10-25

**Authors:** Valeri D Guajardo, Bruno PF Souza, Sérgio G Henriques, Mara CS Lucia, Paulo R Menezes, Milton A Martins, Leila SLPC Tardivo, Wagner F Gattaz, Renério Fráguas

**Affiliations:** 1 Department of Psychiatry, Institute of Psychiatry of Clinics Hospital Faculty of Medicine University of Sao Paulo, (Rua Dr. Ovídio Pires de Campos, 785 - 3° andar - sala 13), Sao Paulo, (CEP: 05403-010), Brazil; 2 Division of Psychology, Central Institute of Clinics Hospital Faculty of Medicine University of Sao Paulo, (Rua Dr. Ovídio Pires de Campos, 225. PAMB Térreo), Sao Paulo, (CEP: 05403-000), Brazil; 3 Department of Preventive Medicine, Faculty of Medicine University of Sao Paulo, (Av. Dr. Arnaldo, 455 - 2° andar), Sao Paulo, (CEP: 01246-903), Brazil; 4 Department of Clinical Medical, Faculty of Medicine University of Sao Paulo, (Av. Dr. Arnaldo, 455 - 1° andar - sala 1216), Sao Paulo, (CEP: 01246-903), Brazil; 5 Department of Clinical Psychology, Institute of Psychology of University of Sao Paulo, (Av. Professor Mello Moraes, 1721 - Bloco F - Cidade Universitária), Sao Paulo, (CEP: 05508-030), Brazil

## Abstract

**Background:**

Depressive symptoms and chronic disease have adverse effects on patients' health-related quality of life (H-RQOL). However, little is known about this effect on H-RQOL when only the two core depressive symptoms - loss of interest and depressed mood - are considered. The objective of this study is to investigate H-RQOL in the presence of loss of interest and depressed mood at a general medical outpatient unit.

**Methods:**

We evaluated 553 patients at their first attendance at a general medical outpatient unit of a teaching hospital. H-RQOL was assessed with the *Medical Outcomes Study 36-item Short-Form Health Survey *(SF-36). Depressed mood and loss of interest were assessed by the *Primary Care Evaluation of Mental Disorders *(PRIME-MD)-Patient Questionnaire. A physician performed the diagnosis of chronic diseases by clinical judgment and classified them in 13 possible pre-defined categories. We used multiple linear regression to investigate associations between each domain of H-RQOL and our two core depression symptoms. The presence of chronic diseases and demographic variables were included in the models as covariates.

**Results:**

Among the 553 patients, 70.5% were women with a mean age of 41.0 years (range 18-85, SD ± 15.4). Loss of interest was reported by 54.6%, and depressed mood by 59.7% of the patients. At least one chronic disease was diagnosed in 59.5% of patients; cardiovascular disease was the most prevalent, affecting 20.6% of our patients. Loss of interest and depressed mood was significantly associated with decreased scores in all domains of H-RQOL after adjustment for possible confounders. The presence of any chronic disease was associated with a decrease in the domain of vitality. The analysis of each individual chronic disease category revealed that no category was associated with a decrease in more than one domain of H-RQOL.

**Conclusion:**

Loss of interest and depressed mood were associated with significant decreases in H-RQOL. We recommend these simple tests for screening in general practice.

## Background

Depression is an important public health problem associated with impairment in quality of life, high levels of disability, increased medical comorbidity and mortality rates [1,2]. In primary care, major depression has a high prevalence ranging from 2.6% to 29.5%, according to epidemiological studies [3-5].

The *Medical Outcomes Study *highlighted the evidence of the adverse effects of depression on patient functional status and well-being [6,7], and various studies have reported the relevant impairment of health-related quality of life (H-RQOL) associated with depression [8-10].

Actually, there is a close association between depression and various domains of H-RQOL [11]; depression has been associated with a decrease in experiencing positive well-being [12], impairment in role functioning [13] and disabilities in social functioning [14]. In fact, several studies show a reduction of H-RQOL in patients suffering from depression as compared with the general population [15,16], as well as with patients with chronic diseases [6,15]. It has also been reported that depressed patients have more substantial and longer-lasting decrements in multiple domains of functioning and well-being than patients with chronic diseases [17].

Depression is frequently associated with chronic disease with a reported 12-month prevalence among patients with chronic disease ranging from 7.9% to 17% [18]. Major depression in patients with chronic disease has been associated with increased symptom burden, which leads to functional impairment, self-care impairment [19], diminished adherence to treatment, undermined general perception of health and reduced levels of energy, social interaction and motivation, consequently decreasing H-RQOL [20]. Studies have shown that chronic diseases can be debilitating and affect a patient's behavior, mood, work capacity, and vital functions, such as ambulation, mobility and the capacity to perform daily activities and eventually H-RQOL [21,22]. These associations are relevant because chronic disease may potentially work as a confounding variable in studies focusing on the negative impact of depression on H-RQOL.

Lately, there has been increased interest in the development, validation and clinical utilization of quick screening and brief scales for depression [23-27]. According to this approach, previous studies have supported that two symptoms, depressed mood and anhedonia (loss of interest), may be enough for detecting depression in primary care [23]. In line with this approach, the aim of our study was to investigate the association between two core symptoms of depression (loss of interest and depressed mood) and H-RQOL in general medical patients taking into account the presence of comorbid chronic disease.

## Methods

### Setting

A cross-sectional survey was conducted in a general internal medicine outpatient unit of a teaching hospital (Ambulatório Geral e Didático da Clínica Médica do Hospital das Clínicas, University of Sao Paulo School of Medicine; AGD - HC-FMUSP) [28,29]. This unit accepts patients from a catchment area of the city of São Paulo and patients referred by other services. Candidate patients are submitted to a screening interview and those with established severe medical conditions are usually referred to an appropriated specialized outpatient unit. During the period from 1998 to 2000, 587 consecutive patients going through their first attendance in that unit were assessed.

### Study population

We included patients of both genders, aged 18 years or older that were going through their first attendance in this hospital's unit. Patients unable to communicate, with severe cognitive deficits or unable to participate in the interview were excluded. We excluded 34 patients because of incomplete data. The remaining 553 patients formed the sample of this study.

Patients were evaluated from Monday to Friday during the morning and afternoon. They were approached and informed about the scope of the interview. The patients that agreed to participate had to read and sign the written consent.

This study was approved and carried out in accordance with ethical standards set by the Ethics Committee of the HC-FMUSP and the Department of Psychiatry, Faculty of Medicine of the University of Sao Paulo.

### Questionnaire

Health-related quality of life was assessed by the *Medical Outcomes Study item-36 Short-Form Health Survey *(SF-36) designed by Ware and Sherboune [30] and validated for the Brazilian-Portuguese by Ciconelli [31]. This questionnaire evaluates the state of physical and mental health individually. The questionnaire consists of 36 items and covers eight domains: physical functioning (PF), role limitations due to physical problems (RP), bodily pain (BP), general health (GH), vitality (VT), social functioning (SF), role limitations due to emotional problems (RE) and mental health (MH). The scores vary from 0 to 100, and a lower score is associated with a worse perception of health, loss of function and presence of pain, and a higher score is associated with a good perception of health, preserved function and no pain.

The SF-36 was designed to be a self-administered instrument, but it can be applied with the assistance of a trained person. In this study, the patient answered the questions in the waiting room assisted by a psychologist.

The assessment of loss of interest and depressed mood were assessed by questions 17 and 18 from the *Patient's Questionnaire of Prime-Md *(QP), which corresponds to loss of interest in routine activities and depressed mood, respectively [29].

The assessment of morbidity was performed by clinicians who filled a questionnaire with yes/no answers for the presence of chronic diseases. Using clinical judgment, clinicians classified the diseases according to 13 predefined categories of disease, including cardiovascular, infectious, endocrine, neurological, rheumatological, gastrointestinal, psychiatric, dermatological, orthopedic, gynaecological, oncological, allergic/immunological or other chronic diseases.

### Statistical methods

The SF-36 scores were calculated according to the established scoring algorithms for the Brazilian version of the SF-36 [31]. The assessment of anhedonia, depressed mood and chronic diseases were reported as absent or present. In addition, the sample was analyzed in three groups including those without chronic disease (n = 225), those with one chronic disease (n = 260) and those with two or more chronic diseases (n = 68) to investigate a potential gradient effect.

We used multiple linear regression to investigate associations between each domain of H-RQOL and our two core depression symptoms. The presence of chronic diseases and demographic variables were included in the models as covariates.

The confidence interval was 95% and significance level was set at p ≤ .05. All statistical analyses were carried out using SPSS for Windows (version 15.0; SPSS Inc., Chicago, IL, USA).

## Results

Among the 553 patients, 70.5% were women and the mean age was 41.0 years (range 18-85, SD ± 15.4). The assessment of depressive symptoms showed that 54.6% of the patients presented loss of interest and 59.7% presented depressed mood. A total of 329 (59.5%) patients had at least one of the thirteen categories of chronic diseases, with cardiovascular the most common affecting 114 (20.6%) patients. The demographic characteristics of the sample are shown in Table 1.

**Table 1:** Demographics, characteristics and prevalence of depressive symptoms and chronic disease

Characteristics	
Age (M ± SD)	41.0 ± 15.4
	
**Gender**	
Female, n (%)	390 (70.5)
Male, n (%)	163 (29.5)
	
**Marital status, n (%)**	
Single	133 (24.1)
Married	281 (50.8)
Divorced	57 (10.3)
Widowed	40 (7.2)
No information	42 (7.6)
	
**Depressive symptoms, n (%)**	
Loss of interest	302 (54.6)
Depressed mood	330 (59.7)
	
**Chronic Disease, n (%)**	329 (59.5)
Cardiovascular	114 (20.6)
Infectious	10 (1.8)
Endocrine	45 (8.1)
Neurological	29 (5.2)
Rheumatological	25 (4.5)
Gastrointestinal	42 (7.5)
Psychiatric	12 (2.1)
Dermatological	20 (3.6)
Orthopedic	19 (3.4)
Gynaecological	16 (2.8)
Oncological	3 (0.3)
Allergic/Immune	26 (4.7)
Other chronic diseases	55 (9.9)

M: mean; SD: standard deviation.

The mean values obtained by the whole sample in SF-36 are listed in Table 2. RP had the lowest score (mean = 32.6) while PF had the highest (mean = 65).

**Table 2:** Domains of SF-36

Domains of SF-36	M ± SD
PF	65.0 ± 26.5
RP	32.6 ± 40.2
BP	42.5 ± 25.7
GH	57.4 ± 24.5
VT	44.3 ± 23.8
SF	64.9 ± 30.0
RE	43.2 ± 42.8
MH	48.8 ± 24.7

M: mean; SD: standard deviation; SF-36: Short Form Health Survey. PF: physical functioning; RP: role functioning physical; BP: bodily pain; GH: general health perceptions; VT: vitality; SF: social functioning; RE: emotional role functioning; MH: mental health

Table 3 shows the univariate analysis with means values and association of domains of SF-36 with presence or absence of loss of interest, depressed mood and chronic diseases. The presence of loss of interest and depressed mood were significantly associated with all domains of the SF-36, while the presence of chronic diseases was associated with a decrease in the domain vitality. In the investigation of a potential gradient effect of chronic disease, the group without chronic diseases showed significantly better scores in the domains PF, RP, VT and RE. The group with one chronic disease was significantly associated with lower scores in VT and the group with two or more chronic disease was significantly associated with lower scores in PF, RP and GH. The results of the multi-variable linear regression are shown in Table 4, which show the constant for each domain of H-RQOL for the all sample and the respective changes for each variable. Loss of interest was associated to a reduction of 7.4 points in PF's score, 26.7 points in RP, 8.8 points in BP, 9.3 points in GH, 15.3 points in VT, 18.9 in SF, 17.6) in RE and 11.7 points in MH. Depressed mood showed association with a decrease of 6.6 points in PF's score, 9.8 points in RP, 8.6 points in BP, 13.2 points in GH, 15.1 points in VT, 15.9 points in SF, 19.4 points in RE and 23.4 points in MH. The presence of a chronic disease was associated with a decrease of 4.6 points in VT's score. This analysis showed that male sex was significantly associated with higher scores in PF, RP, BP, VT and MH. Patients with age >65 years had the highest RE scores, and single patients presented the highest scores in PF, RP and RE.

**Table 3:** Mean values and association of domains of SF-36 with presence or absence of depressive symptoms and chronic disease

	PF	RP	BP	GH	VT	SF	RE	MH
All sample (M ± SD)	64.8 ± 26.5	32.6 ± 40.2	42.5 ± 25.7	57.4 ± 24.5	44.3 ± 23.8	64.8 ± 30.1	43.2 ± 42.8	49.7 ± 24.7
Loss of interest (95% CI)	59.7 ± 25.8 [6.7;15.5]	18.5 ± 31.4 [24.7;37.5]	36.7 ± 23.5 [8.5;17.0]	50.8 ± 23.5 [10.5;18.3]	34.6 ± 19.6 [17.8;25.0]	53.6 ± 29.3 [20.1;29.2]	31.5 ± 39.4 [18.9;32.7]	39.4 ± 21.3 [16.8;24.4]
Without loss of interest (95% CI)	70.9 ± 26.2 [6.7;15.4]	49.7 ± 43.0 [24.9;37.3]	49.5 ± 26.5 [8.6;16.9]	65.3 ± 23.3 [10.5;18.3]	56.0 ± 23.2 [17.8;25.0]	78.3 ± 25.2 [20.0;29.2]	57.3 ± 42.6 [18.9;32.7]	60.0 ± 23.9 [16.8;24.4]
Depressed mood (95% CI)	60.5 ± 26.2 [6.2;15.1]	24.2 ± 35.7 [14.0;27.8]	37.0 ± 23.3 [9.2;18.0]	50.5 ± 23.8 [13.1;20.8]	35.6 ± 20.5 [17.8;25.3]	55.4 ± 29.7 [18.5;27.7]	32.4 ± 39.4 [19.8;33.9]	37.3 ± 19.8 [24.9;32.0]
Without depressed mood (95% CI)	71.2 ± 25.9 [6.2;15.1]	45.1 ± 43.1 [14.3;27.5]	50.7 ± 27.0 [9.4;17.9]	67.5 ± 21.9 [13.0;20.8]	57.2 ± 22.7 [17.9;25.2]	78.6 ± 24.8 [18.4;27.9]	59.3 ± 42.6 [19.9;33.8]	65.7 ± 21.3 [25.0;31.9]
Chronic disease (95% CI)	61.0 ± 26.6 [5.0;13.8]	28.5 ± 39.4 [3.3;16.9]	42.1 ± 25.9 [-3.2;5.5]	56.0 ± 23.3 [-0.7;7.7]	41.8 ± 23.2 [2.0;10.1]	63.5 ± 30.8 [-1.9;8.1]	40.1 ± 41.9 [0.4;15.1]	48.2 ± 24.8 [-2.7; 5.6]
Without chronic disease (95% CI)	70.4 ± 25.4 [5.0;13.9]	38.7 ± 40.6 [3.3;16.9]	43.2 ± 25.5 [-3.2;5.5]	59.5 ± 26.0 [-0.6;7.6]	47.9 ± 24.4 [2.0;10.1]	66.6 ± 29.0 [-1.9;8.2]	47.9 ± 43.8 [0.5;15.0]	49.6 ± 24.7 [-2.7; 5.6]

The values shown represent the mean, standard deviation and confidence interval (95% CI). M: mean; SD: standard deviation; SF-36: Short Form Health Survey. PF: physical functioning; RP: role functioning physical; BP: bodily pain; GH: general health perceptions; VT: vitality; SF: social functioning; RE: emotional role functioning; MH: mental health.

**Table 4:** Multiple linear regressions for the association of domains of SF-36 with depressive symptoms, clinical and socio-demographic variables

	PF	RP	BP	GH	VT	SF	RE	MH
Constant	70.9	47.4	49.0	70.7	62.9	85.4	60.6	67.4
Gender (95% CI)	+8.8*** [4.4;13.2]	+7.0* [0.4;13.7]	+10.8*** [6.4;15.2]	-	+6.7*** [3.1;10.4]	-	-	+5.8** [2.2;9.4]
Categorized age 1 (18-24 years)	-	-	-	-	-	-	-	-
Categorized age 2 (25-44 years)	-	-	-	-	-	-	-	-
Categorized age 3 (45-64 years)	-	-	-	-	-	-	-	-
Categorized age 4 (>65 years)	-	-	-	-	-	-	+9.8	-
Single (95% CI)	+14.9*** [10.3;19.5]	+14.7*** [7.6;21.8]	-	-	-	-	+12.3** [4.4;20.1]	-
Loss of interest (95% CI)	-7.4** [-11.6;-3.1]	-26.7*** [-33.2;-20.1]	-8.8*** [-13.1;-4.5]	-9.3*** [-13.3;-5.2]	-15.3*** [-18.8;-11.7]	-18.9*** [-23.7;-14.1]	-17.6*** [-24.8;-10.4]	-11.7*** [-15.2;-8.1]
Depressed mood (95% CI)	-6.6** [-11.0;-2.3]	-9.8** [-16.5;-3.1]	-8.6*** [-13.1;-4.2]	-13.2*** [-17.3;-9.0]	-15.1*** [-18.8;-11.5]	-15.9*** [-20.7;-11.0]	-19.4*** [-26.7;-12.1]	-23.4*** [-27.0;-19.8]
Chronic disease (95% CI)	-	-	-	-	-4.6** [-8.0;-1.2]	-	-	-
Cardiovascular disease (95% CI)	-12.1*** [-17.0;-7.2]	-	-	-	-	-	-	-
Endocrine disease (95% CI)	-	-	+10.7** [3.4;18.0]	-	-	-	-	-
Rheumatological disease (95% CI)	-	-	-12.4* [-22.1;-2.8]	-	-	-	-	-
Psychiatric disease (95% CI)	-	-	-	-14.9* [-27.9;-1.9]	-	-	-	-
Dermatological disease (95% CI)	-	-	-	-	-	-15.2* [-27.0;-3.3]	-	-
Orthopedic disease (95% CI)	-	-	-	-	-11.4* [-20.6;-2.2]	-	-	-
Gynaecological disease (95% CI)	-17.1** [-28.9;-5.2]	-	-	-	-	-	-	-
Oncological disease (95% CI)	-39.9** [-66.7;-13.1]	-	-	-	-	-	-	-
Other chronic diseases (95% CI)	-11.1** [-17.7;-4.5]	-	-	-		-	-	-

The values shown represent the regression coefficient and confidence interval (95% CI). One equation was constructed for each domain of SF-36 as the dependent variable and the depressive symptom, clinical and socio-demographic variable as the independent variable. Each equation was adjusted for variables found to be associated with the respective domain in the bi-variate analysis. PF: physical functioning; RP: role functioning physical; BP: bodily pain; GH: general health perceptions; VT: vitality; SF: social functioning; RE: emotional role functioning; MH: mental health.* *P *< 0.05, ** *P *< 0.01, *** *P *< 0.001.

## Discussion

In this study of 553 general practice outpatients, we found associations between decreased H-RQOL and two simple screening measures for depression (loss of interest and depressed mood). Both symptoms were independently associated with lower scores on all subscales of the SF-36. These results are consistent with past reports of the association between depression and H-RQOL [8,19,32]. Previous studies have investigated the association of H-RQOL with depressive symptoms' scales or with the diagnosis of depression. On this point, our study differs in that we investigated whether only two self-reported screening symptoms for depression were associated with decreased H-RQOL. Because the evaluation of loss of interest and depressed mood are quick, inexpensive and non-invasive and, as shown by our study, are significantly associated with low H-RQOL, we suggest they are useful indicators of H-RQOL in general practice.

Our data reinforce previous studies supporting the use of these two symptoms in screening for major depressive disorder among medical patients. One study with multiple sclerosis patients found that the presence of these two symptoms could identify almost all patients with major depressive disorder, with minimal numbers of false positive [24]. Another study that used the Beck Depression Inventory II (BDI-II) to detect major depressive disorder, concluded that one or two questions regarding sadness and loss of interest serve as simple and effective screening tools for depression post-acute myocardial infarction [33].

An intriguing point of our results is the association of depressed mood and loss of interest with the various domains of the SF-36. Previous studies have reported impaired quality of life in patients with depression, especially when compared to the general population and patients with other chronic diseases [34], such as diabetes, arthritis or cardiovascular disease [6,8,17,35]. It should be noted that the SF-36 includes two domains, the physical one formed by PF, RP, BP and GH and the mental one formed by VT, SF, RE and MH. If we apply this reasoning, our data revealed that the two depressive symptoms affect both the physical and the mental domains, while chronic diseases affect almost exclusively the physical domains. Similar results were described in a study of patients with differing chronic diseases in Germany that compared the H-RQOL in patients of general practice with the general population [22]. Actually, in our study, no specific chronic disease affected more than one domain of SF-36. A similar pattern has been reported by a Chinese study, in which the presence of a chronic disease was associated with impairment of some but not all domains of H-RQOL [36]. The main difference between these studies and ours is that we focused only on two depressive symptoms.

Approximately seventy percent of patients visiting the AGD are female and this proportion is similar to other studies that assessed the H-RQOL in primary care such as Fleck et al (2002) [32] (79.5%) and Schwenk et al (1996) [37] (79.8%). Women's higher demand for the health service in our study may have been influenced by the association found between depressed mood and female gender (p = 0.02), since there wasn't any association between chronic disease and gender. Alternatively, it is possible that the increased demand by women for health services is related to their decreased H-RQOL in five of the eight domains of the SF-36, PF, RP, BP, VT and MH, but we cannot establish a causality due to the cross-sectional nature of our study.

In our sample, 12% of the patients visiting the AGD had more than one of the thirteen chronic diseases. This prevalence was relatively low compared with those reported in the literature. The proportion of multi-morbidity (two or more medical conditions) in general practice patients varies in previous studies with Wang et al (2008) [22] reporting it in 20%, van den Akker et al (1998) [38] in 29.7% and Lam and Lauder (2000) [36] in 41% of the population under study. However, it is difficult to compare these studies because of many differences in methodology, target population, and the number and type of the diseases under study.

Some limitations of our study should be considered. The assessment of chronic disease was made by clinical judgment without validated instruments, and this might have introduced bias into our results. Furthermore, the severity of the chronic diseases was not assessed. However, we performed our analyses using three groups, those without chronic disease, those with one chronic disease and those with two or more chronic disease. In this analysis we found a gradient effect showing that those with two or more chronic disease presented with lower scores in PF, RP and GH while those with one chronic disease presented with lower scores only in VT. Although a gradient effect could be supported, it should be noted that even for those with two or more chronic disease the association with lower scores on H-RQOL was not as pervasive as it was for depressed mood and loss of interest. In addition, this study did not evaluate the impact of the treatment of depression on H-RQOL. It is also appropriate to mention that this was a cross-sectional study and cannot establish causality between associations. Last, while the study suggests that loss of interest and depressed mood are indicative of low H-RQL, it should be remembered that these items are not sufficient to diagnose clinical depression and cannot replace a comprehensive clinical assessment of mood in the context of chronic disease.

## Conclusion

This study indicates that loss of interest and depressed mood are associated with decreased H-RQOL. Loss of interest and/or depressed mood were associated with lower scores in all eight domains of SF-36, while each chronic disease affected no more than one domain of H-RQOL. These findings are relevant because they show how loss of interest and/or depressed mood might affect H-RQOL.

This study also suggests that the assessment of loss of interest and depressed mood would be useful as a screening for low H-RQOL, since these two questions are quick, inexpensive and the presence of at least one of these symptoms has a significant association with reduced scores in all domains of SF-36.

## Competing interests

The authors declare that they have no competing interests.

## Authors' contributions

SGH and RF participated in the design of the Prime-MD project, carried out the study, supervised the data collection, and revised the manuscript. MCSL and LSLPCT participated in the design of the Prime-MD project and carried out the project. PRM, MAM and WFG participated in the design of the Prime-MD project, carried out the project, and discussion of results. VDG and BPFS developed the design of the study, developed the rationale of the statistical analysis and drafted the manuscript. All authors read and approved the final manuscript.

## Pre-publication history

The pre-publication history for this paper can be accessed here:

http://www.biomedcentral.com/1471-2458/11/826/prepub
